# Giant Liver Infections: Cryptogenic Liver Abscess Secondary to Bacteroides Fragilis

**DOI:** 10.7759/cureus.26283

**Published:** 2022-06-24

**Authors:** Hassnain R Syed, Patricia Tellez Watson

**Affiliations:** 1 Internal Medicine, University of Kentucky, Bowling Green, USA; 2 Infectious Diseases, The Medical Center-Bowling Green, Bowling Green, USA

**Keywords:** liver abscess, pyogenic liver abscess (pla), liver abscess drainage, pyogenic hepatic abscess, liver abscess aspiration

## Abstract

Hepatic abscesses are a seldom finding although they are the most common type of intraabdominal abscess. We find that this localized collection of pus can be a result of bacteria, ameba, or fungi. This study will mainly focus on the first type, the pyogenic liver abscess (PLA), in which management is predicated on size of abscess. There is no standard definition as to what constitutes a small or large hepatic abscess. Here, we present a case of a 10 cm PLA found in the right lobe of the liver. We aimed to discuss the relevant epidemiology, pathophysiology, clinical manifestations, and management of PLAs.

## Introduction

A liver abscess usually presents as a single abscess, most commonly invading the right lobe of the liver, however, multiple abscesses can occur. The two most common types of liver abscesses, pyogenic and amebic, have a global distribution; however, in developed countries, pyogenic liver abscesses (PLAs) are far more prevalent, where amebic liver abscesses (ALA) reign supreme in developing regions [1]. Fungal liver abscesses are extremely rare, accounting for less than 2% of cases [2]. Interestingly enough, liver abscesses present with seemingly nonspecific symptoms, hence making the differential quite broad. Patients usually have fever, chills, and right upper quadrant abdominal pain. PLAs account for approximately one to four cases per 100,000 persons annually in the United States [1]. The majority of PLAs are polymicrobial with enteric Gram-negative bacilli, largely *Escherichia coli*, being most prevalent. Among Gram-positive organisms, streptococci and enterococci species are commonly reported, and anaerobic infection with Bacteroides spp. is also a common source of PLA formation [1]. The incidence of PLA cases is dramatically higher in Asia, with most cases being secondary to *Klebsiella pneumoniae* [1]. These cases were first reported in Taiwan in diabetic patients with no hepatobiliary disease. Since the first reported case in the mid-1980s, community-acquired *K. pneumoniae* now accounts for 80% of all PLAs in Asia [3]. Liver abscesses carry a low incidence rate, and rarer still is the "giant" liver abscess- defined by a size of 10 cm or greater. Here, we present a 78-year-old male presenting with fevers and chills, who was found to have a giant PLA.

## Case presentation

Our patient is a 78-year-old male with a significant history of controlled diabetes mellitus type 2, essential hypertension, obstructive sleep apnea, and gastroesophageal reflux disease (GERD) who presented to an outside facility for evaluation of fevers with reported temperature of 104°F.

**Figure 1:**
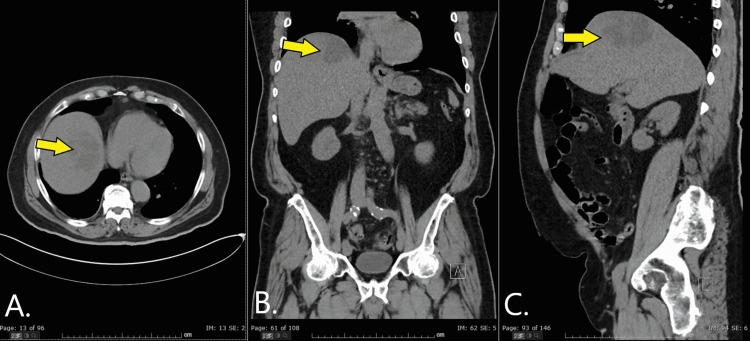
Axial (A), coronal (B), and sagittal views of CT scan abdomen/pelvis without contrast The images show an approximate 8 cm lesion (yellow arrow) in the superior portion of the right hepatic lobe.

He was recently hospitalized one month prior for treatment of coronavirus disease 2019 (COVID-19) pneumonia. During that stay, the patient was reported to have transaminitis, with a subsequent computed tomography (CT) abdomen revealing an approximate 8 cm lesion, noted to be possible necrotic abscess, in the superior portion of the right hepatic lobe (Figure 1). The patient denied symptoms related to the lesion. The hepatic lesion was reportedly treated with intravenous (IV) antibiotics and once respiratory symptoms from COVID-19 were treated, he was discharged home.

As it pertains to his current visit, his review of systems was largely unremarkable, aside from generalized fatigue. Physical examination revealed no abnormal findings. Laboratory workup showed significant leukocytosis at 21.39 k/uL with a neutrophil predominance (86%) on differential and normal liver function tests. A right upper quadrant ultrasound was performed which showed a 9.21 cm hepatic mass in the upper right lobe, indicating growth of this lesion when compared to previous diagnostic imaging. He was treated with IV antibiotics for several days before being transferred to our facility for further care.

**Figure 2:**
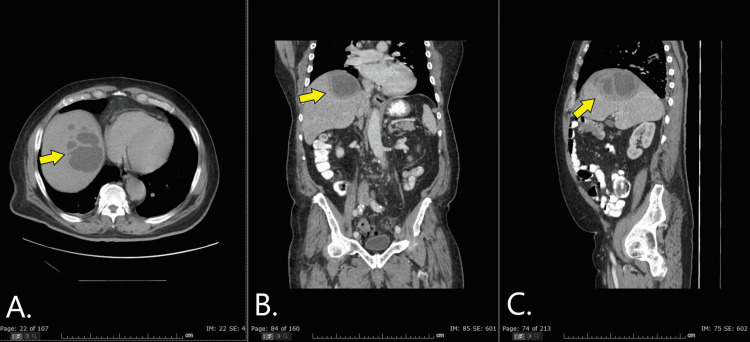
Axial (A), coronal (B), and sagittal (C) views of CT abdomen/pelvis with contrast The images depict the liver lesion one month after initial CT scan - now measuring 10 cm in diameter and with multiple well-defined low-density areas, representing abscess (yellow arrow). Note the characteristic “double target sign” which is best appreciated on the coronal and sagittal views.

Upon arrival at our facility, we obtained a set of blood cultures and antibiotic coverage was started with scheduled cefepime 2 g IV three times a day (TID) and scheduled metronidazole 500 g IV TID. A CT abdomen/pelvis with IV contrast was performed which showed the large liver lesion, now measuring 10 cm in diameter and with multiple well-defined low-density areas likely representing abscess. A “double target sign” could be appreciated this time, i.e., a rim-enhancing fluid collection with surrounding low attenuated outer ring-characteristic of a hepatic abscess (Figure 2). The gallbladder, biliary ducts, appendix, and ileocecal junction appeared normal, with patent portal and hepatic veins. Further history intake did not reveal recent travel nor contact with anyone with recent travel to endemic areas. Social history negative for tobacco abuse, alcohol abuse, or recreational drug use. Initial set of blood cultures showed no growth. 

**Figure 3:**
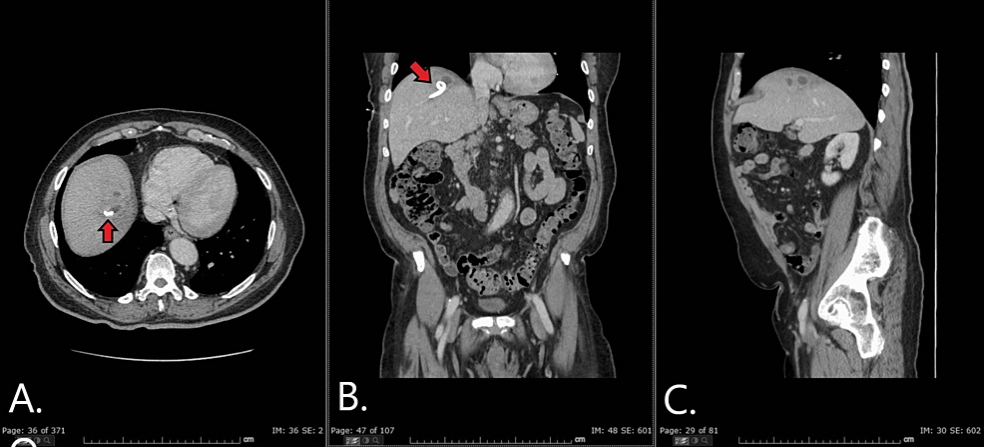
Axial (A), coronal (B), and sagittal (C) views of CT scan abdomen/pelvis with contrast three weeks post-discharge The images show a drastic decrease in abscess size post hepatic drain (red arrow) placement.

The patient was scheduled for percutaneous hepatic drain placement. Aerobic/anaerobic cultures taken from the abscess showed no growth, however, polymerase chain reaction (PCR) performed on the liver abscess aspirate detected DNA from *Bacteroides fragilis*. The patient responded well to this intervention, showing no evidence of fever or leukocytosis. Parenteral antibiotics, now having been administered for one week, were transitioned to oral metronidazole 500 mg TID. The patient was discharged with the intention of obtaining a repeat CT scan abdomen three weeks post discharge to assess abscess size and antibiotic duration. The patient returned to office for his follow-up CT scan which showed a drastic decrease in abscess size (Figure 3). The patient had no acute complaints and no reported fevers at home. Metronidazole 500mg PO TID was continued to complete a six-week total antibiotics course. His hepatic drain was also removed after a total six-week duration. The patient fully recovered with no complications or readmission.

## Discussion

The first case of PLA has been described since the time of the “father of medicine,” Hippocrates. He proposed that the prognosis of a patient correlated with the type of fluid within the abscess cavity [4]. At present we know that a considerable portion of PLAs is formed via bowel leakage that travels to the liver through the portal vein, or portal pyemia [5]. However, the most common cause of PLA is by far direct spread from biliary disease, with cholangitis being the major identifiable etiology [1]. Other precipitating factors include other intraabdominal infections, such as pancreatitis or diverticulitis and trauma. Despite sophistication in diagnostic imaging, cryptogenic PLAs are quite frequent. A PLA is cryptogenic when no extrahepatic source is identified [6]. 

PLAs are usually identified on abdominal imaging. Ultrasound (US) and CT are the common modalities used, with CT usually being employed if US findings are nonspecific. CT is slightly more sensitive than US for liver abscess (95% versus 85%) [7,8]. Peripheral rim enhancement with or without surrounding edema, although uncommon, is specific for liver abscess [7,8]. After obtaining blood cultures, management of a PLA is centered around drainage and empiric antibiotic administration- antibiotic therapy must not be delayed while abscess cultures are waiting to be collected. Antibiotic therapy should cover Gram-negative bacilli, Gram-positive cocci, and anaerobes. Examples of reasonable initial therapy include piperacillin-tazobactam, third-generation cephalosporins (e.g., ceftriaxone) plus metronidazole, or fluoroquinolone (e.g., levofloxacin) plus metronidazole.

We typically treat PLAs for four to six weeks with antibiotics and adequate source control. Source control, in this case abscess drainage, is predicated on abscess size. Generally, abscess size can be divided into two groups, less than or equal to 5 cm (small abscesses) and more than 5 cm (large abscesses) [9]. Small abscesses are usually drained via percutaneous needle aspiration. Large abscesses are drained with percutaneous catheter placement. Catheters remain in place until resolution of clinical signs of infection, daily patent catheter output <10-20 mL, and repeat imaging showing resolution of the fluid collection or reduction in size to <3 cm [10,11]. PLAs are generally treated with parenteral antibiotics for one to three weeks once adequate source control is achieved. Upon signs of clinical improvement (no reported fevers, no leukocytosis, decreased drainage, decreased abscess size on imaging), patients are transitioned to oral antibiotics to complete the remainder of the four to six weeks [1].

There has been debate on the modality used for drainage, especially in regard to larger abscesses. Liao et al. have reported percutaneous drainage failure with abscess size greater than 7.3 cm, recommending surgical drainage for such abscess sizes [12]. A giant PLA has been defined as abscess size greater than or equal to 10 cm [13]. Ahmed et al. performed a retrospective review of all giant PLA patients treated at a tertiary center from 2001 to 2011, which concluded that percutaneous drainage is safe and effective in even giant PLAs, suggesting that large abscess size does not indicate immediate surgical drainage [13]. Most resources agree that surgical drainage is seldom necessary but can be considered under the following circumstances: unsuccessful percutaneous drainage, multiloculated abscesses, or cases of intraperitoneal rupture [9]. Patients' comorbidities also play a role in what type of management is best suited for a PLA, giant or not. For instance, percutaneous and surgical approach might be unsafe in a cirrhotic patient with thrombocytopenia or history of bleeding diathesis. Hayashi et al. described an endoscopic transpapillary abscess drainage technique via nasobiliary tube [14]. This approach limits bleeding complications and also affords simultaneous opportunity to rule out biliary causes of PLAs.

This case serves to remind us of another important fact - obtain abscess PCR along with cultures. Anaerobic bacteria are notorious for being difficult to culture. As in our case, the abscess culture did not grow any bacteria, but we confirmed the presence of *B. fragilis* via PCR and tailored antibiotic management accordingly. This case is unique in that the patient did not have any risk factors or common predisposing pathology for PLA formation. The patient was infected with *B. fragilis*, which is a part of the human gastrointestinal flora, specifically found in the colon [15]. Normal pathophysiology dictates that disruption of the mucosa by means of inflammation or trauma would introduce the microbe into the bloodstream, further perpetuating infection. Oddly enough, the patient's diagnostic workup did not reveal common conditions predisposing to PLAs such as cholangitis, diverticulitis, pancreatitis, appendicitis, or abscess formation via extension from a contiguous focus like the kidney. Hence, this giant PLA was most likely cryptogenic in origin.

## Conclusions

The majority of PLAs are polymicrobial with the most common pathogens being Gram-negative bacilli (largely *E. coli*), Gram-positive cocci (largely streptococci and enterococci), and anaerobes (largely Bacteroides spp.) Right upper quadrant US and CT are the common modalities used for diagnosis, with CT being slightly more sensitive than US for liver abscess. Management of a PLA is centered around source control drainage and empiric antibiotic administration. All PLAs should be treated with antibiotics for four to six weeks with adequate source control drainage. Drainage modalities are determined by abscess size. Large abscesses, including giant PLAs, can adequately be drained via percutaneous methods and seldom require surgical intervention.
